# Trends in hospital volume and operative mortality in hepato-biliary surgery in Veneto region, Italy

**DOI:** 10.1007/s13304-023-01574-9

**Published:** 2023-07-03

**Authors:** Alfredo Guglielmi, Marzia Tripepi, Laura Salmaso, Ugo Fedeli, Andrea Ruzzenente, Mario Saia

**Affiliations:** 1 Department of Surgery, Division of General and Hepatobiliary Surgery, School of Medicine, University of Verona, Piazzale L. Scuro, 10, 37123 Verona, Italy; 2 Azienda Zero, Veneto Region, Padua, Italy

**Keywords:** Volume outcomes, Hepatobiliary surgery, Centralization

## Abstract

Hepatobiliary resections are among the most complex and technically challenging surgical procedures. Even though robust evidence showed that complex surgical procedures such as hepatobiliary surgery have better short- and long-term outcomes and lower mortality rate when performed in high-volume centers, the minimal criteria of centers that can perform hepatobiliary activity are not clearly defined. We conducted a retrospective population study of patients who underwent hepatobiliary surgery for malignant disease in a single Italian administrative region (Veneto) from 2010 to 2021 with the aim to investigate the hospitals annual surgical volume for hepatobiliary malignant diseases and the effect of hospital volume on in-hospital, 30- and 90-day postoperative mortality. The centralization process of hepatobiliary surgery in Veneto is rapidly increasing over the past 10 years (rate of performed in highly specialized centers increased from 62% in 2010 to 78% in 2021) and actually it is really established. The crude and adjusted (for age, sex, Charlson Index) mortality rate after hepatobiliary surgery resulted significantly lower in centers with high-volume activity compared to them with low-volume activity. In the Veneto region, the “Hub and Spoke” model led to a progressive centralization of liver and biliary cancer treatment. High surgical volume has been confirmed to be related to better outcomes in terms of mortality rate after hepatobiliary surgical procedures. Further studies are necessary to clearly define the minimal criteria and associated numerical cutoffs that can help define the characteristics of centers that can perform hepatobiliary activities.

**Supplementary Information:**

The online version contains supplementary material available at 10.1007/s13304-023-01574-9.

## Introduction

During the past decades, worldwide, increasing efforts have been made to increase the centralization of health care in high-volume hospital for complex and risky procedures such as surgery for hepatobiliary cancers.

Implementing centralization is seen as an opportunity to increase the quality of care, based on the principle that experience and high specialization resulting from the high number of patients treated, are related with an improvement of quality of health assistance and patient’s surgical outcome [1].

Hepatobiliary resections are among the most complex and technically challenging surgical procedures and require an accurate selection of candidates to surgery, a preoperative precise surgical planning involving multidisciplinary team, high technical surgical skill, an accurate anesthesiologic management, and specialized postoperative care in case of postoperative complications.

Robust evidence shows that complex surgical procedures such as hepatobiliary surgery have better short- and long-term outcomes and lower mortality rate when performed in high-volume centers; specifically high hospital volume is frequently associated with better indication to surgery, patients’ selection and improved preoperative optimization, higher surgical expertise, as well as optimization of the management of postoperative complications with lower failure to rescue [1–6].

However, despite that there is a general consensus on the importance of centralization for hepatobiliary surgery, the determinants involved in this process (including a minimum annual caseload of liver surgery) are not clearly defined.

In a previous review article on centralization of liver surgery in Italy, Torzilli et al. have defined minimal hospital requirements for surgical units that should be entitled to perform liver surgery in Italy and included, among others, a surgical volume requirement of more than 20 liver resections per year for malignant liver diseases with a 90-day mortality rate < 3% [7].

The aim of this reports was to investigate the annual surgical volume for hepatobiliary malignant diseases during a 11 years’ time period (2010–2021) for hospitals of a single Italian administrative region (Veneto, northeastern Italy, 4.9 milion residents). Moreover, the effect of hospital volume on in-hospital, 30- and 90-day postoperative mortality was analyzed.

## Methods

This was a retrospective population study of patients who underwent liver surgery for malignant disease in a single Italian administrative region (Veneto) from 2010 to 2021.

Data were obtained from the anonymized archive of hospital discharge forms (“schede di dimissione ospedaliera – SDO)” whose database includes information on inpatient discharge from every single hospital stay at any public or private accredited hospital. The Regional Healthcare Service collects information on all hospital admissions (elective and emergency) recorded using hospital discharge forms (SDO), which are based on the International Classification of Diseases, Ninth Revision, Clinical Modification (ICD-9-CM) classification. Data collected using SDO included the following: patient demographics (gender, date/place of birth, and place of residence), admission details (date of admission, identification code of the ward/hospital of admission), discharge data (discharge date, main diagnosis, and up to 5 secondary diagnoses, main intervention and up to 5 secondary procedures with relevant dates), and diagnosis-related groups.

The population registry includes the date of death of residents of the Veneto region; This database was used to establish overall mortality at 30 days and 90 days from the date of surgery, while SDO were used to analyze intra-hospital mortality.

### Inclusion criteria

All admissions between 2010 and 2021 in any public or private accredited hospital in Veneto, with at least one of the diagnoses of primary and secondary malignant tumor of the liver or bile ducts (ICD-9-CM: 155.0, 155.1, 155.2, 156.0, 156.1, 156.2, 156.8, 156.9, 197.7) and at least one of the major hepato-biliary (HB) surgical procedures (ICD-9-CM: 50.21, 50.22, 50.23, 50.24, 50.25, 50.29, 50.3, 50.4, 50.5, 50.61, 50.69,51.22,51.23, 51.63, 51.69, 51.36, 51.37, 51.39, 51.94) were included into the study.

### Exclusion criteria

Patients age < 18 years or > 100 years; moreover, distal pancreatectomy, pancreaticoduodenectomy and total pancreatectomy (intervention ICD9CM code 52.5, 52.6, 52.7) were excluded.

#### Definitions

The total number of hepatobiliary procedures performed during the study in each hospital was recorded.

According to surgical volume, hospital has been distinct as follows:

- Very-low volume hospital: < 5 surgical hepato-biliary procedures per year for malignant disease
- Low-volume hospital: 5–19 surgical hepato-biliary procedures per year for malignant disease
- Intermediate volume hospital: 20–99 surgical hepato-biliary procedures per year for malignant disease
- High specialized hospital: ≥ 100 surgical hepato-biliary procedures per year for malignant disease.

Since the classification is based on the annual surgical volume, over the entire analysis period, the same hospital may result in different groups in different years.

Surgical procedures have been classified in five groups as follows:

1. Liver transplant (intervention ICD9CM code: 50.5).
2. Liver resection (intervention ICD9CM code: 50.3, 50.4, 50.22).
3. Biliary procedures (intervention ICD9CM code: 51.22, 51.23, 51.63, 51.69, 51.36, 51.37).
4. Liver ablation (intervention ICD9CM code: 50.23, 50.24, 50.25).
5. Other procedures (intervention ICD9CM code: 50.21, 50.29, 50.61, 50.69, 51.39, 51.94).

In case of more than one surgical procedure from different groups performed during the same hospitalization, the above hierarchical order has been applied.

Mortality has been investigated as in-hospital mortality, 30- and 90- days postoperative mortality.

In order to assure a complete follow-up for mortality analysis, subjects who did not reside in Veneto region or who had a prior hospitalization for liver malignant disease (diagnosis ICD9CM code: 155, 197.7, V10.07) in a period among 5 years and 6 months before the hospitalization or who underwent a liver lobectomy or total hepatectomy in the five years before surgery (intervention ICD9CM code: 50.3, 50.4) were excluded from the mortality analysis.

### Statistical analysis

First, a descriptive analysis of the annual trend of overall hospitalizations for liver surgery in Veneto has been conducted and afterwards stratification was made according to type of intervention and class of annual surgical volume.

Moreover, a descriptive analysis has been conducted on the annually relationship between hospital volume, type of surgical resection, and in-hospital, 30 -and 90-day postoperative mortality. Cochran-Armitage test of trend was used to examine the presence of a trend across the study period as regards in-hospital, 30- and 90-day postoperative mortality. A *p* < 0.05 was considered statistically significant.

Hierarchical logistic regression models were used to account for the multilevel structure of the data and to adjust for clustering of patients within hospitals. The association of hospital volume with mortality outcomes was estimated by odds ratio after controlling for patients’ characteristics (age, gender and comorbidities measured by the Charlson index).

All statistical analyses were conducted using SAS (Statistical Analysis System) software version 9.4 (SAS Institute Inc., Cary, NC, USA).

## Results

### Hospital volume

During the study period, 17,159 hospitalizations for liver or bile duct malignancy with at least one surgical procedure has been recorded, with an average of 1400 hepato-biliary surgeries per year.

Among the entire cohort of patients who underwent surgery, 4945 patients (28.8%) reside in Veneto region. Except for the first year (20.9%) with lower numbers compared to data of the following years, a growing number of patients residing outside the region was submitted to surgery, from 27.2 to 31.5%. (Table 1).

**Table 1:** Number of HB surgeries according to year of admission and patient’ residence

Year of discharge	Residence		Total
	Outside of Veneto region *N* (%)	Veneto region *N* (%)	
2010	259 (20.9)	982 (79.1)	1241
2011	378 (27.2)	1011 (72.8)	1389
2012	300 (25.2)	889 (74.8)	1189
2013	367 (27.7)	957 (72.3)	1324
2014	399 (28.4)	1008 (71.6)	1407
2015	458 (29.8)	1080 (70.2)	1538
2016	489 (31.5)	1063 (68.5)	1552
2017	478 (30.3)	1099 (69.7)	1577
2018	461 (30.3)	1061 (69.7)	1522
2019	502 (31.7)	1084 (68.3)	1586
2020	453 (31.0)	1008 (69.0)	1461
2021	401 (29.2)	972 (70.8)	1373
Total	4945 (28.8)	12,214 (71.2)	17,159

Almost all surgical hepato-biliary procedures (92.7%) were performed in public hospitals. (Supplementary Table 2).

In 2010 and 2011 the frequency of surgical procedures performed in accredited private hospitals was, respectively, 17% and 21% but starting from 2012 we observed a reduction in the activity carried out in these hospitals, with values of 5% in the more recent period. Although until 2015, there was no significant variation in the number of private accredited structures involved (attesting between 7 and 9 structures), in recent years, procedures have been performed in only 4 private accredited hospitals.

The most frequently performed procedures were liver resection (39.8%) and liver ablation (36.6%), followed by biliary procedures (9.2%), and liver transplantation (3.9%) (Table 2).

**Table 2:** Number of surgeries according to type of surgical procedures across years

Year	Surgical procedure											
	Liver transplant		Liver resection		Biliary procedures		Liver ablation		Other		Total	
	*N*	%	*N*	%	*N*	%	*N*	%	*N*	%	*N*	%
2010	26	2.1	482	38.8	132	10.6	284	22.9	317	25.5	1241	100
2011	39	2.8	549	39.5	155	11.2	412	29.7	234	16.9	1389	100
2012	51	4.3	458	38.5	138	11.6	394	33.1	148	12.5	1189	100
2013	39	3.0	569	43.0	141	10.7	460	34.7	115	8.7	1324	100
2014	51	3.6	566	40.2	141	10	543	38.6	106	7.5	1407	100
2015	54	3.5	572	37.2	142	9.2	607	39.5	163	10.6	1538	100
2016	67	4.3	554	35.7	136	8.8	645	41.6	150	9.7	1552	100
2017	82	5.2	599	38.0	142	9.0	619	39.3	135	8.6	1577	100
2018	74	4.9	566	37.2	120	7.9	635	41.7	127	8.3	1522	100
2019	65	4.1	640	40.4	126	7.9	638	40.2	117	7.4	1586	100
2020	62	4.2	676	46.3	97	6.6	542	37.1	84	5.8	1461	100
2021	54	3.9	602	43.9	106	7.7	502	36.6	109	7.9	1373	100
Total	664	3.9	6833	39.8	1576	9.2	6281	36.6	1805	10.5	17,159	100

Definition: liver transplant (intervention ICD9CM code:, 50.5); Liver resection (intervention ICD9CM code: 50.3, 50.4, 50.22); Bile duct resection (intervention ICD9CM code: 51.63, 51.69, 51.36, 51.37, 51.22, 51.23); Liver ablation (intervention ICD9CM code: 50.23, 50.24, 50.25); Other (intervention ICD9CM code: 50.21, 50.29, 50.61, 50.69, 51.39, 51.94)

The majority of interventions (69.2%; *n* = 11,882) were performed in hospitals with at least 100 hepatobiliary surgeries per year for malignant disease. In detail, 3 high-volume centers performed more than 60% of the total number of surgeries; moreover, the rate of hepatobiliary procedures performed in high-volume centers increased over the time from 62.1% (2010) to 78.0% (2021) (*p*-value for trend: < 0.0001).

Centers with intermediate volume (20–99 hepato-biliary procedures per year) performed 18.4% (*n* = 3.165) of surgeries with a significant reduction in the past years, from 20.7% (2010) to 14.2% (2021).

Finally, only 10% (*n* = 1712) of surgical activity was performed in low-volume center (5–19 hepatobiliary procedures per year for malignant disease) and 2.3% (*n* = 400) in center with very low volume of hepatobiliary procedures, although this group included a large number of hospitals [*n* = 18 (2010); *n* = 14 (2021)] (Table 3).

**Table 3:** Number of hospitals and surgeries according to procedural volume and year. Veneto, years 2010–2021

Year	Hospital volume (Hepato-biliary procedures per year)												Total		
	100 + operation			20–99 operation			5–19 operation			0–4 operation					
	H (*N*)	Admission (*N*)	%	H (*N*)	Admission (*N*)	%	H (*N*)	Admission (*N*)	%	H (*N*)	Admission (*N*)	%	H (*N*)	Admission (*N*)	%
2010	3	770	62.1	7	257	20.7	16	181	14.6	18	33	2.7	44	1241	100
2011	3	866	62.4	6	284	20.5	18	199	14.3	17	40	2.9	44	1389	100
2012	2	706	59.4	7	259	21.8	16	179	15.1	19	45	3.8	44	1189	100
2013	2	851	64.3	7	260	19.6	16	171	12.9	16	42	3.2	41	1324	100
2014	2	864	61.4	11	375	26.7	12	132	9.4	15	36	2.6	40	1407	100
2015	2	982	63.9	8	343	22.3	16	179	11.6	16	34	2.2	42	1538	100
2016	2	1056	68.0	9	341	22.0	13	129	8.3	10	26	1.7	34	1552	100
2017	3	1176	74.6	10	296	18.8	8	76	4.8	13	29	1.8	34	1577	100
2018	3	1156	76.0	5	180	11.8	13	155	10.2	15	31	2.0	36	1522	100
2019	3	1210	76.3	7	227	14.3	13	124	7.8	13	25	1.6	36	1586	100
2020	3	1174	80.4	6	148	10.1	9	107	7.3	15	32	2.2	33	1461	100
2021	3	1071	78.0	7	195	14.2	8	80	5.8	14	27	2.0	32	1373	100
Total	–	11,882	69.2	–	3.165	18.4	–	1.712	10	–	400	2.3	–	17,159	100

Definition: H: hospital; N = number

According to the type of surgical interventions, liver transplantation was performed only in two high-volume centers and rate of liver ablation were higher in high volume compared to intermediate, low and very low volume; 41.1% (*n* = 4880), 32% (*n* = 1013), 19.6% (*n* = 335) and 13.3% (*n* = 53), respectively (Table 4).

**Table 4:** Number of surgeries according to type of surgical procedures and hospital surgical volume

Procedure	Hospital volume (Hepato-biliary procedures per year)									
	100 + procedures		20–99 procedures		5–19 procedures		0–4 procedures		Total	
	*N*	%	*N*	%	*N*	%	*N*	%	*N*	%
Liver transplant	664	5.6	0	0,0	0	0.0	0	0.0	664	3.9
Liver resection	4867	41.0	1082	34.2	748	43.7	136	34.0	6833	39.8
Biliary procedures	714	6.0	351	11.1	366	21.4	145	36.3	1576	9.2
Liver ablation	4880	41.1	1013	32.0	335	19.6	53	13.3	6281	36.6
Others	757	6.4	719	22.7	263	15.4	66	16.5	1805	10.5
Total	11,882	100	3.165	100	1.712	100	400	100	17,159	100

Veneto, years 2010–2021

Definition: liver transplant (intervention ICD9CM code: 50.5, 50.4,); liver resection (intervention ICD9CM code: 50.3, 50.22); bile duct resection (intervention ICD9CM code: 51.63, 51.69, 51.36, 51.37, 51.22, 51.23); liver ablation (intervention ICD9CM code: 50.23, 50.24, 50.25); other (intervention ICD9CM code: 50.21, 50.29, 50.61, 50.69, 51.39, 51.94)

### Type of hepatobiliary procedures

During the period in exam, among a total of 17,159 hepatobiliary surgical procedures performed, 8777 (51.2%) have been performed for patients affected by hepatocarcinoma (HCC). Among this patients, 2440 (27.8%) were also affected by cirrhosis.

The majority of patient with HCC (*n* = 6799, 77.5%) and of them with HCC and cirrhosis (*n* = 1693, 69.4%) has been treated at high-volume hospital.

Conversely, the rate of HCC patients and HCC with cirrhosis patients who underwent hepatobiliary surgical procedures was around 5–8% in low-volume hospital and lower than 1% in very low-volume hospital. (Supplementary Table 3) Stratifying patients according to the diagnosis of HCC, we found a heterogeneity of surgical approach among different hospital volume. In detail, while in High specialized hospital and Intermediate volume hospital the rate of liver resection and liver ablation for patients with HCC was similar, in low- and very-low volume hospital the rate of liver resection was extensively higher than that of liver ablation. (Supplementary Table 3).

In detail, patients with HCC underwent predominantly liver ablation in high-volume hospitals (57.2%, *n* = 3888) while the predominant treatment for these patients in very low-volume centers was liver resection (42.6%, *n* = 23).

This tendency has been confirmed also for patients with HCC and cirrhosis with a rate of 28.8% of liver resection and of 37.7% of liver ablation performed in high volume hospital and a rate of 42.1% and 36.8% of respectively liver resection and liver ablation performed in very-low volume hospital. (Supplementary Table 3).

### Minimally invasive liver resection

Among a total of 6833 liver resection intervention (ICD9CM code: 50.3, 50.22) performed during the study period in Veneto, the rate of those performed by minimally invasive approach (intervention ICD9CM code: 50.21) was 23.6%. The percentage of liver resection performed by minimally invasive approach increased over time (from 8% in 2010–2013 to 39.5% in 2018–2021).

According to hospital volume, high-volume hospitals showed the higher rate of minimally invasive approach with a frequency of 42% in the last period (2018–2021) compared to 17.5% of very-low volume hospitals. (Supplementary Table 4).

### Mortality

From an initial cohort of 17,159 patients, after exclusion of patients not residing in Veneto region and who had a liver lobectomy or hepatectomy in the previous 5 years, the mortality analysis was performed on 7989 (46.6%) patients.

In the examined period, the in-hospital, 30- and 90-day postoperative mortality were 1.8% (*n* = 147), 1.9% (*n* = 150) and 5.3% (*n* = 422), respectively. In-hospital mortality declined over time (from 2.2% in 2010–2013 to 1.4% in 2018–2021 p-value for trend: 0.0167); conversely, the 30-day mortality (p-value for trend: 0.118) and 90-day mortality (p-value for trend: 0.379) showed similar values over the study period. (Table 5).

**Table 5:** Distribution of in-hospital, 30-day, and 90-day mortality of patients resident in Veneto according to procedural volume by time period

Mortality	Hospital volume (procedure per year)														
	Total			100+			20–99			5–19			0–4		
	*N*	Deaths	%	*N*	Deaths	%	*N*	Deaths	%	*N*	Deaths	%	*N*	Deaths	%
*In-hospital*															
2010–2013	2618	57	2.2%	1239	19	1.5%	677	12	1.8%	558	23	4.1%	144	3	2.1%
2014–2017	2772	54	1.9%	1455	24	1.6%	786	10	1.3%	422	16	3.8%	109	4	3.7%
2018–2021	2599	36	1.4%	1718	15	0.9%	450	13	2.9%	333	8	2.4%	98	0	0.0%
TOT	7989	147	1.8%	4412	58	1.3%	1913	35	1.8%	1313	47	3.6%	351	7	2.0%
*30-day*															
2010–2013	2618	53	2.0%	1239	19	1.5%	677	9	1.3%	558	20	3.6%	144	5	3.5%
2014–2017	2772	56	2.0%	1455	21	1.4%	786	11	1.4%	422	17	4.0%	109	7	6.4%
2018–2021	2599	41	1.6%	1718	17	1.0%	450	11	2.4%	333	9	2.7%	98	4	4.1%
TOT	7989	150	1.9%	4412	57	1.3%	1913	31	1.6%	1313	46	3.5%	351	16	4.6%
*90-day*															
2010–2013	2618	135	5.2%	1239	55	4.4%	677	29	4.3%	558	37	6.6%	144	14	9.7%
2014–2017	2772	158	5.7%	1455	75	5.2%	786	32	4.1%	422	40	9.5%	109	11	10.1%
2018–2021	2599	129	5.0%	1718	72	4.2%	450	27	6.0%	333	19	5.7%	98	11	11.2%
Total	7989	422	5.3%	4412	202	4.6%	1913	88	4.6%	1313	96	7.3%	351	36	10.3%

Veneto, years 2010–2021

According to hospital volume, the postoperative outcomes were slightly lower in high-volume hospitals compared to intermediate volume hospital as follows: in-hospital mortality of 1.3% and 1.8%; 30-day mortality of 1.3% and 1.6% and 90-day mortality of 4.6% and 4.6%. Conversely, low and very low volume centers showed higher in hospital mortality (3.6% and 2.0%), 30-day mortality (3.5% and 4.6%) and 90-day mortality (7.3% and 10.3%).

Multivariable analysis was performed to confirm the role of hospital volume for postoperative outcomes adjusted for confounders (age, gender, Charlson index), specifically postoperative mortality was significantly higher in low- and very-low volume hospital with an OR of 2.65 (95% CI 1.69–4.17) and 3.42 (95% CI 1.85–6.32) for 30-day mortality and 1.55 (95% CI 1.19–2.02) and 2.17 (95% CI 1.47–3.19) for 90-day mortality. These results have been confirmed when analysis was limited to major surgical procedures excluding liver ablations (*N* = 5843); in particular, postoperative mortality was significantly higher in low and very-low volume hospital with an OR of 2.49 (95% CI 1.53–4.05) and 3.12 (95% CI 1.64–5.94) for 30-day mortality and 1.48 (95% CI 1.10–2.00) and 2.09 (95% CI 1.39–3.15) for 90-day mortality (Table 6).

**Table 6:** Association of hospital volume with in-hospital, 30-day and 90-day mortality: crude odds ratios (OR), and adjusted estimates obtained by multilevel logistic regression models

a. All hepato-biliary procedures												
Hospital volume (hepato-biliary procedures per year) *N* = 7989	In hospital mortality				30-day postoperative mortality				90-day postoperative mortality			
	Crude OR (95% CI)	*p* value	Adj OR (95% CI)*	*p* value	Crude OR (95%CI)	*p* value	Adj OR (95%CI)*	*p* value	Crude OR (95%CI)	*p* value	Adj OR (95%CI)*	*p* value
100+	Rif		Rif		Rif		Rif		Rif		Rif	
20–99	1.38 (0.82;2.33)	0.2275	1.28 (0.75;2.18)	0.363	1.27 (0.78;2.09)	0.341	1.17 (0.71;1.93)	0.5349	1.00 (0.77;1.31)	0.982	0.93 (0.71;1.23)	0.6223
5–19	2.79 (1.72;4.52)	< 0.0001	2.66 (1.63;4.34)	< 0.0001	2.82 (1.81;4.41)	< 0.0001	2.65 (1.69;4.17)	< 0.0001	1.64 (1.27;2.13)	0.0002	1.55 (1.19;2.02)	0.0012
0–4	1.51 (0.65;3.5)	0.3394	1.42 (0.61;3.34)	0.420	3.67 (2.01;6.72)	< 0.0001	3.42 (1.85;6.32)	< 0.0001	2.38 (1.63;3.48)	< 0.0001	2.17 (1.47;3.19)	< 0.0001

b. Excluded patients who underwent liver ablation (intervention ICD9CM code: 50.23, 50.24, 50.25)												
Hospital volume (liver resection per years)	In hospital mortality				30-day postoperative mortality				90-day postoperative mortality			
	Crude OR (95%CI)	*p* value	Adj OR (95%CI)*	*p* value	Crude OR (95%CI)	*p* value	Adj OR (95%CI)*	*p* value	Crude OR (95%CI)	*p* value	Adj OR (95%CI)*	*p* value
100+	Rif		Rif		Rif		Rif		Rif		Rif	
20–99	1.21 (0.69;2.13)	0.5009	1.11 (0.62;2.00)	0.717	1.27 (0.73;2.19)	0.401	1.16 (0.67;2.02)	0.6042	1.00 (0.73;1.37)	0.9866	0.93 (0.68;1.29)	0.6688
5–19	2.30 (1.39;3.80)	0.0012	2.16 (1.28;3.65)	0.004	2.68 (1.66;4.33)	< 0.0001	2.49 (1.53;4.05)	0.0003	1.61 (1.2;2.15)	0.0015	1.48 (1.10;2.00)	0.0098
0–4	1.19 (0.51;2.80)	0.6929	1.11 (0.46;2.66)	0.820	3.36 (1.79;6.3)	0.0002	3.12 (1.64;5.94)	0.0005	2.36 (1.58;3.52)	< 0.0001	2.09 (1.39;3.15)	0.0004

*Adj OR: adjusted OR for age, sex, Charlson Index

Moreover, stratifying patients according to diagnosis, patients with HCC and those with HCC and cirrhosis who underwent liver resection showed a higher in-hospital, 30-day and 90-day postoperative mortality when compared with patients without HCC/cirrhosis although this difference was not statistically significant. In detail, the greatest risk of postoperative mortality for HCC and cirrhosis patients compared with other patients was identified 90 days after surgical liver resection (90-day mortality HCC plus cirrhosis 5.3% vs 3.9% patients with other malignant liver tumor, crude OR 1,35, 95% CI 10.85; 2.16). (Supplementary Table 5).

## Discussion

Although previous studies have examined the role of hospital resection volume in outcome in HB surgery, this is the first Italian regional study that showed the state of the centralization process of liver surgery in the past 10 years in Veneto and tested the association between hospital volume and operative mortality.

After the introduction of the Veneto Oncological Network in 2013, a group of experts have defined the diagnostic and therapeutic pathways (“percorsi diagnostici terapeutici e assistenziali-PDTA”) for oncological hepatobiliary disease with the aim to implement good clinical practices and define the most suitable organizational models to give to patient the best level of care [8].

The goal of this project was to structurally centralize patients from local low-volume hospital (“Spoke”) to a central high-volume hospital (“Hub”) optimizing available resources and reducing waste.

According to the results of this study, although a specific regional administrative law has not been introduced, the centralization process of liver surgery in Veneto is rapidly increasing over the past 10 years (rate of liver resection performed in highly specialized centers increased from 62.1% in 2010 to 78.0% in 2021) and actually it is really established.

This study showed either the increasing role of high-volume hospital in the treatment of patients with hepatobiliary disease and the significant differences in mortality rate after hepatobiliary surgery between centers with low-volume and high-volume activity.

These results are consistent with previous literature, suggesting the relationship of hospital volume with the occurrence of postoperative mortality [9, 10]. The reasons that could explain the correlation between high hospital volume and better surgical outcomes are several, including a more specialized medical team, a standardization of care and a higher experience with treatment of complications [2, 11–14].

Interestingly, we found similar results for mortality after HB surgery at high (≥ 100 surgical hepato-biliary procedures per year) and intermediate (20–99 surgical hepato-biliary procedures per year) hospital volume.

In detail, the in-hospital and 30-day mortality rate in high- and intermediate-volume hospital was satisfying and similar to data reported in literature (< 2%) [15]. Conversely both low- and very-low volume hospital reported a significantly high 30-day mortality (3.6% and 4.6%, respectively).

Similarly, compared with a 90-day mortality of less than 5% in high- and intermediate-volume hospitals, this value reaches around 7–10% in low- and very-low volume hospitals.

After adjusting for age, sex, Charlson Index, the difference among in-hospital, 30-day and 90-day postoperative mortality between high- and intermediate-volume hospital was not significantly different, while there was around a 2–threefold increase of postoperative mortality both for low-volume (5–19) and very-low volume hospital (0–4) as in terms of 30-day mortality (OR 2.65 and 3.47 respectively) as in terms of 90-day mortality (OR 1.64 and OR 2.38, respectively) when compared to high-volume hospital.

These results have been confirmed also when it has been considered only hepatobiliary resection after exclusion of liver ablation.

According with these results, there is an evident benefit in term of postoperative mortality reduction above a surgical volume of 20 annual hepatobiliary resections per year. Despite that there is not yet established a minimal volume requirement for a Hepatobiliary surgical unit, the cut-off of 20 liver resection per year is commonly accepted in several countries as discriminant for acceptable postoperative outcome [7, 16–18].

In particular, Dimick et al. in a study on more than 2,000 hepatectomies performed in North America found that those hospitals that performed more than 20 liver resections per year had significantly lower mortality rate (3.9% vs 7.6% at low-volume hospitals (*p* < 0.001). This result has been confirmed at the multivariate analysis where high-volume hospitals showed a 40% lower risk of in-hospital mortality compared with low-volume hospitals (OR, 0.60; 95% CI 0.39–0.92; *p* = 0.02] [19].

These results have been confirmed also for minimally invasive liver surgery (MILS); Van der Poel et al., in a multicentric study on 6951 liver resections performed, of which 916 were MILS (13%), showed that centers which performed ≥ 20 MILS annually had less conversions and less overall complications (37 (30%) versus 58 (42%) of center with < 20 MILS per year, *p* = 0.040) [18].

Furthermore, the range of difficulty for hepatobiliary procedures may vary significantly, especially among different diagnoses and patients with associated comorbidities. For these reasons, a minimal annual volume should also consider parameters related to the level of complexity. This approach could be interesting for optimizing resources and improving outcomes through the centralization of the most complex resections in highly experienced centers. Simultaneously, it would direct simpler cases to intermediate and low-volume centers. With the aim to better stratify the surgical risk, we investigate the distribution of high-risk patients such as those with cirrhosis among the different hospitals according to hospital volume.

We confirmed that liver cirrhosis represents a risk for postoperative mortality and complications including ascites, jaundice, postoperative liver failure and encephalopathy also for minor liver resections. HCC with cirrhosis was associated with an increased mortality risk, especially for 90-day postoperative mortality even if without statistically significant probably due to the paucity of data after sample stratification. For these reasons, liver resections in patients with cirrhosis still represent a medical challenge and require an accurate preoperative risk evaluation and a careful surgical and postoperative management. According to our results, these complex patients has been predominantly treated at high- and intermediate-volume hospital which may have technical equipments and dedicated multidisciplinary teams allowing a more effective preoperative surgical planning and a better management of postoperative complications [9].

Interestingly, when analyzing the type of surgical approach among hospitals of different volumes, we found that patients with HCC predominantly underwent liver ablation in high-volume hospitals, while liver resection was more common in very-low volume hospitals. This difference may be attributed to lower familiarity with ultrasound-guided procedures or a limited availability of dedicated interventional liver-directed services in low and very-low volume hospitals.

Another interesting point of this study is the distribution of minimally invasive approach according to hospital volume. In recent years, minimally invasive liver resection gained a key role in the surgical treatment of malignant and benign liver tumors and there are robust evidences that minimally invasive liver resection is associated with less intraoperative blood loss, early oral feeding, fewer complications, shorter postoperative hospital stay and similar oncological outcomes compared to open liver resections (OLR) [20, 21] According to literature, this study confirmed that minimally invasive surgery is increasing over the time (from 8% in 2010–2013 to 39.5% in 2018–2021). However, because of its complexity requiring expertise in both the laparoscopic and liver surgery, it is predominantly performed in high-volume hospital (42% compared to 17.5% of very-low volume hospital in the late period 2018–2021).

The results of this study should be analyzed in light of limitations, in particular the use administrative data to analyze clinical limits the number of available variables. Clearly, the description of mortality rate according to hospital volume represents a first step of a more complex and detailed exploration that is necessary to analyze factors related with outcomes. For example, the intervention ICD9CM coded as 50.22 refers generally to “Partial liver resection” without distinction between minor and major hepatectomy (> 3 liver segment) [22].

In addition, several new technical approaches have been recently proposed to achieve parenchymal sparing hepatectomies, usually more complex than major hepatectomies: in these cases the classic distinction between major and minor hepatectomies is inadequate to describe the complexity of surgery [23, 24].

Another limit of the study is that it was not possible to analyze the failure to rescue (FTR), that means mortality after experiencing a surgical complication. The FTR is considered a quality indicator that focuses on management of complications rather than on mortality itself [25].

A recent Italian study focusing on FTR within 90 days after resection of HCC observed a significant lower FTR rate in high volume hospital (5.1%) compared to low-volume hospital (28.6%) [9].

Several studies reported the importance of a timely and appropriate treatment of patients with a major complication after hepato-biliary surgery. The lower FTR in high hospital volume seems to be related to be a better expertise and to the availability of a multidisciplinary approach, including among others an experienced anesthesiologist and intensive care unit, a interventional radiologist and endoscopist, a hepatologist, as well as a nutritional and nurse team [9, 26, 27].

In conclusion, this study showed that in the Veneto region, the Hub and Spoke model led to a progressive centralization of liver and biliary cancer treatment.

Moreover, this study has confirmed that a high surgical volume is associated with better outcomes in terms of mortality rates. However, further studies are necessary to clearly define the minimal criteria and numerical cutoffs that can help identify the characteristics of centers more qualified of performing hepatobiliary procedures. It is important to note that these criteria may vary depending on the specific country or region, and local healthcare authorities and experts should be consulted to ensure compliance with local regulations and standards.

Further studies are needed to comprehend all the factors associated with higher mortality rates and failure-to-rescue rates. The aim is to refine the centralization process and improve healthcare for patients with hepatobiliary diseases. This centralization involves a complex evaluation and the development of dedicated policies to promote high-quality care and optimize human and technological resources.

Concurrently, this process requires continuous monitoring of outcomes to ensure that quality standards are met throughout the therapeutic and diagnostic phases for patients with hepatobiliary diseases.

Additionally, the growing rate of patients seeking care outside the region demonstrates that the centralization of hepatobiliary care extends beyond the Veneto region. However, this "health migration" incurs significant economic and time costs for patients and their families. Therefore, policies are needed to facilitate the centralization process and reduce the burden on patients when accessing the best therapeutic treatments for their health conditions.

### Supplementary Information

Below is the link to the electronic supplementary material.

Supplementary file1 (DOCX 30 KB)
