# Supplementary material for: Trends in hospital volume and operative mortality in hepato-biliary surgery in Veneto region, Italy
Source: Updates Surg. 2023 Jul 3;75(7):1949–59. doi: 10.1007/s13304-023-01574-9 (PMC10543584; doi:10.1007/s13304-023-01574-9)
Supplement: Supplementary file 1 — Supplementary file1 (DOCX 30 KB) [file 13304_2023_1574_MOESM1_ESM.docx]

**Supplementary Tab. 1.** Diagnosis and Intervention code (ICD-9 CODE) included in the analysis

| **DIAGNOSIS CODE (ICD-9 CODE)** | **INTERVENTION CODE (ICD-9 CODE)** |
| --- | --- |
| 155.0 Primary liver neoplasm | 50.21 Marsupialization of lesion of liver |
| 155.1 Malignant neoplasm of intrahepatic bile ducts | 50.22 Partial hepatectomy |
| 155.2 Malignant liver neoplasm not specified as primary or secondary | 50.23 Open ablation of liver lesion or tissue |
| 156.0 Malignant neoplasm of gallbladder | 50.24 Percutaneous ablation of liver lesion or tissue |
| 156.1 Malignant neoplasm of extrahepatic bile duct | 50.25 Laparoscopic ablation of liver lesion or tissue |
| 156.2 Malignant neoplasm of Ampulla of Vater | 50.29 Other destruction of lesion of liver |
| 156.8 Malignant neoplasm of other specified sites of gallbladder and extrahepatic bile ducts | 50.3 Lobectomy of liver |
| 156.9 Malignant neoplasm of biliary tract, part unspecified | 50.4 Total hepatectomy |
| 197.7 Malignant liver neoplasm specified as secondary | 50.5 Liver transplantation |
|  | 50.61 Closure of laceration of liver |
|  | 50.69 Other repair of liver |
|  | 51.22 Cholecystectomy |
|  | 51.23 Laparoscopic cholecystectomy |
|  | 51.63 Other excision of common duct |
|  | 51.69 Excision of other bile duct |
|  | 51.36 Choledocho-enterostomy |
|  | 51.37 Anastomosis of hepatic duct to gastrointestinal tract |
|  | 51.39 Other bile duct anastomosis |
|  | 51.94 Revision of anastomosis of biliary tact |

**Supplementary Tab. 2** Number of hospitals and surgeries per years according to type of hospital (Public or Private Accredited hospital)

| **Year** | **Public Hospital** | | | **Private Accredited Hospital** | | | **Total** | | |
| --- | --- | --- | --- | --- | --- | --- | --- | --- | --- |
|  | **H (N)** | **Admission** | **%** | **H (N)** | **Admission** | **%** | **H (N)** | **Admission** | **%** |
| 2010 | 36 | 1027 | 82,8 | 8 | 214 | 17,2 | 44 | 1241 | 100 |
| 2011 | 36 | 1097 | 79,0 | 8 | 292 | 21,0 | 44 | 1389 | 100 |
| 2012 | 35 | 1109 | 93,3 | 9 | 80 | 6,7 | 44 | 1189 | 100 |
| 2013 | 33 | 1249 | 94,3 | 8 | 75 | 5,7 | 41 | 1324 | 100 |
| 2014 | 33 | 1316 | 93,5 | 7 | 91 | 6,5 | 40 | 1407 | 100 |
| 2015 | 33 | 1462 | 95,1 | 9 | 76 | 4,9 | 42 | 1538 | 100 |
| 2016 | 29 | 1468 | 94,6 | 5 | 84 | 5,4 | 34 | 1552 | 100 |
| 2017 | 27 | 1497 | 94,9 | 7 | 80 | 5,1 | 34 | 1577 | 100 |
| 2018 | 31 | 1449 | 95,2 | 5 | 73 | 4,8 | 36 | 1522 | 100 |
| 2019 | 31 | 1516 | 95,6 | 5 | 70 | 4,4 | 36 | 1586 | 100 |
| 2020 | 29 | 1414 | 96,8 | 4 | 47 | 3,2 | 33 | 1461 | 100 |
| 2021 | 28 | 1298 | 94,5 | 4 | 75 | 5,5 | 32 | 1373 | 100 |
| **Total** | **-** | **15902** | **92,7** | **-** | **1257** | **7,3** | **-** | **17159** | **100** |

**Supplementary Tab. 3** Number of surgeries for patients with Hepatocarcinoma (HCC) and cirrhosis according to type of surgical procedures and hospital surgical volume. Veneto, years 2010-2021

a. Patients with Hepatocarcinoma (HCC)

| **Procedure** | **Hospital volume (Hepato-biliary procedures per years)** | | | | | | | | | | |
| --- | --- | --- | --- | --- | --- | --- | --- | --- | --- | --- | --- |
|  | **100+ procedures** | | **20-99 procedures** | | **5-19 procedures** | | **0-4 procedures** | | **Total** | | **P value** |
|  | **N** | **%** | **N** | **%** | **N** | **%** | **N** | **%** | **N** | **%** | <.0001 |
| Liver transplant | 638 | 9,4 | 0 | 0,0 | 0 | 0,0 | 0 | 0,0 | 638 | 7,3 |  |
| Liver resection | 1768 | 26,0 | 345 | 23,5 | 156 | 34,3 | 23 | 42,6 | 2292 | 26,1 |  |
| Liver ablation | 3888 | 57,2 | 685 | 46,6 | 206 | 45,3 | 19 | 35,2 | 4798 | 54,7 |  |
| Others | 505 | 7,4 | 439 | 29,8 | 93 | 20,5 | 12 | 22,3 | 1049 | 11,9 |  |
| **Total** | **6799** | **100** | **1469** | **100** | **455** | **100** | **54** | **100** | **8777** | **100** |  |

b. Patients with Hepatocarcinoma (HCC) and cirrhosis

| **Procedure** | **Hospital volume (Hepato-biliary procedures per years)** | | | | | | | | | | |
| --- | --- | --- | --- | --- | --- | --- | --- | --- | --- | --- | --- |
|  | **100+ procedures** | | **20-99 procedures** | | **5-19 procedures** | | **0-4 procedures** | | **Total** | | **P value** |
|  | **N** | **%** | **N** | **%** | **N** | **%** | **N** | **%** | **N** | **%** | <.0001 |
| Liver transplant | 444 | 26,2 | 0 | 0,0 | 0 | 0,0 | 0 | 0,0 | 444 | 18,2 |  |
| Liver resection | 487 | 28,8 | 139 | 26,4 | 60 | 29,9 | 8 | 42,1 | 694 | 28,4 |  |
| Liver ablation | 639 | 37,7 | 158 | 30,0 | 84 | 41,8 | 7 | 36,8 | 888 | 36,4 |  |
| Others | 123 | 7,3 | 240 | 43,7 | 57 | 28,4 | 4 | 21,1 | 414 | 17,0 |  |
| **Total** | **1693** | **100** | **527** | **100** | **201** | **100** | **19** | **100** | **2440** | **100** |  |

**Supplementary Tab. 4** Distribution of minimally invasive liver resection (intervention ICD9CM code: 50.3, 50.22) according to procedural volume by time period. Veneto, years 2010-2021

|  | **Hospital volume**  **(Hepatobiliary procedures/year)** | **Total Liver resection** | **Minimally invasive liver resection** | |
| --- | --- | --- | --- | --- |
| **Period** | **N** | **N** | **N** | **%** |
| 2010-2013 | 100+ | 1238 | 90 | 7.3 |
|  | 20-99 | 441 | 21 | 4.7 |
|  | 05-19 | 327 | 51 | 15.6 |
|  | 00-04 | 52 | 2 | 3.8 |
|  | Total | 2058 | 164 | 7.9 |
|  | | | | |
| 2014-2017 | 100+ | 1587 | 353 | 22.3 |
|  | 20-99 | 437 | 76 | 17.4 |
|  | 05-19 | 223 | 41 | 18.4 |
|  | 00-04 | 44 | 2 | 4.5 |
|  | Total | 2291 | 472 | 20.6 |
|  | | | | |
| 2018-2021 | 100+ | 2042 | 858 | 42 |
|  | 20-99 | 204 | 54 | 26.5 |
|  | 05-19 | 198 | 62 | 31.3 |
|  | 00-04 | 40 | 7 | 17.5 |
|  | Total | 2484 | 981 | 39.5 |
| 2010-2021 |  | 6833 | 1617 | 23.6 |

**Definition: Liver resection intervention (ICD9CM code: 50.3, 50.22); Minimally invasive liver resection (ICD9CM code 54.21)**

**Supplementary Tab. 5** Association of Hospital Volume with in-hospital, 30-day and 90-day mortality: crude odds ratios (OR) for liver resection in patients with HCC and HCC plus cirrhosis

|  |  | Postoperative Mortality | | | | | |
| --- | --- | --- | --- | --- | --- | --- | --- |
| Diagnosis | Total Liver resection | In hospital | | 30-day mortality | | 90-day mortality | |
|  |  | N | % | N | % | N | % |
| HCC with cirrhosis | 433 | 9 | 2.1 | 7 | 1.6 | 23 | 5.3 |
| HCC without cirrhosis | 881 | 18 | 2 | 20 | 2.3 | 42 | 4.8 |
| Other | 2434 | 42 | 1.7 | 33 | 1.4 | 97 | 3.9 |
| Total | 3748 | 69 | 1.8 | 60 | 1.6 | 162 | 4.3 |

|  | Postoperative Mortality | | | | | |
| --- | --- | --- | --- | --- | --- | --- |
| Diagnosis | In hospital | | 30-day mortality | | 90-day mortality | |
|  | Crude OR | (95%CI) | Crude OR | (95%CI) | Crude OR | (95%CI) |
| HCC with cirrhosis | 1.2 | 0.58; 2.50 | 1.2 | 0.53; 2.72 | 1.4 | 0.85; 2.16 |
| HCC without cirrhosis | 1.2 | 0.68; 2.08 | 1.7 | 0.97; 2.96 | 1.2 | 0.83; 1.75 |
| Other | Ref. | - | Ref. | - | Ref. | - |
